# Low-Intensity Focused Ultrasound-Mediated Attenuation of Acute Seizure Activity Based on EEG Brain Functional Connectivity

**DOI:** 10.3390/brainsci11060711

**Published:** 2021-05-27

**Authors:** Minjian Zhang, Bo Li, Xiaodong Lv, Sican Liu, Yafei Liu, Rongyu Tang, Yiran Lang, Qiang Huang, Jiping He

**Affiliations:** 1 School of Mechatronical Engineering, Beijing Institute of Technology, Beijing 100081, China; 3120170166@bit.edu.cn (M.Z.); 3120170125@bit.edu.cn (B.L.); liu_sican@bit.edu.cn (S.L.); yafei.liu@bit.edu.cn (Y.L.); qhuang@bit.edu.cn (Q.H.); JIPING.HE@asu.edu (J.H.); 2 Beijing Advanced Innovation Center for Intelligent Robots and Systems, Beijing Institute of Technology, Beijing 100081, China; xiaodong.lv@bit.edu.cn (X.L.); yiran.lang@bit.edu.cn (Y.L.)

**Keywords:** ultrasound, EEG, epilepsy, brain network, synchronization

## Abstract

(1) Background: Ultrasound has been used for noninvasive stimulation and is a promising technique for treating neurological diseases. Epilepsy is a common neurological disorder, that is attributed to uncontrollable abnormal neuronal hyperexcitability. Abnormal synchronized activities can be observed across multiple brain regions during a seizure. (2) Methods: we used low-intensity focused ultrasound (LIFU) to sonicate the brains of epileptic rats, analyzed the EEG functional brain network to explore the effect of LIFU on the epileptic brain network, and continued to explore the mechanism of ultrasound neuromodulation. LIFU was used in the hippocampus of epileptic rats in which a seizure was induced by kainic acid. (3) Results: By comparing the brain network characteristics before and after sonication, we found that LIFU significantly impacted the functional brain network, especially in the low-frequency band. The brain network connection strength across multiple brain regions significantly decreased after sonication compared to the connection strength in the control group. The brain network indicators (the path length, clustering coefficient, small-worldness, local efficiency and global efficiency) all changed significantly in the low-frequency. (4) Conclusions: These results revealed that LIFU could reduce the network connections of epilepsy circuits and change the structure of the brain network at the whole-brain level.

## 1. Introduction

Ultrasound is widely used in physiotherapy and medical diagnostics. Ultrasound neuromodulation for brain stimulation does not require surgery or genetic alteration, but it provides superior spatial resolution and depth penetration compared to other noninvasive methods. Transcranial ultrasound can modulate neuronal activity [1,2], neural network connections [3], and cerebral hemodynamics [4,5,6]. Ultrasound has been shown to reduce the occurrence of seizure EEG bursts and the severity of epileptic behavior [7]. Ultrasound stimulation can also inhibit spontaneous recurrent seizures in the acute period of epilepsy and improve performance of behavioral tests evaluating depression and sociability during chronic epilepsy [8].

Epilepsy is a transient functional disorder of the brain caused by sudden abnormal discharge of brain neurons and can cause short-term obstacles of consciousness and behavior in patients [9]. Drug therapy and surgical treatment are the two main anti-epilepsy strategies. However, most antiepileptic drugs have obvious toxic side effects, and many tests suggest that they seriously damage liver and kidney function. Surgery has precise and demanding requirements for preoperative evaluation and exact localization of epileptic focus and preoperative assessment [10]. Epilepsy surgery may cause cerebral edema, intracranial hematoma, infection of surgical wounds, occurrence of skull osteomyelitis and so on [11]. Therefore, it is imperative to find a high-accuracy epilepsy treatment method without side effects. Traditional studies show that the epileptic focus is the region where epilepsy begins and serves as the target of surgical intervention [12]. From the perspective of the epileptic circuit, the formation of a single epileptic focus cannot necessarily cause a seizure, and the epileptic circuit is the physical and physiological basis of seizures [13]. This indicates that seizures might be a more complex network and that different brain regions play different roles in the evolution of epilepsy. Brain functional connectivity provides more important information than simply analyzing the changes in neuronal activity in the local brain region, complex network theory is a very powerful tool that can reveal the mechanism and characteristics of brain structure and function that have not been revealed by past analysis methods.

The brain is a highly complex system often undergoing numerous interactions and that has topological properties, represented as a structurally interconnected network by a dense of cortico-cortical axonal pathways and a functionally synchronized network by external or intrinsic coherent neural activity [14,15]. Existing research on complex brain networks based on neuroimaging, such as electroencephalography (EEG), magnetoencephalography (MEG), functional magnetic resonance imaging (fMRI) and diffusion tensor imaging (DTI), has shown that complex network theory is a very powerful tool that can reveal the mechanism and characteristics of brain structure and function that have not been revealed by past analysis methods [16,17,18]. In 2007, Ponten constructed brain function networks of EEG signals in different stages of seizures and found that the characteristic path length increased during and after the onset of epilepsy [19]. Adi used microelectrode arrays to evaluate EEG changes in the hippocampus of chemically induced epileptic rats during a seizure episode and found that the local network of the hippocampus showed first desynchronization and then synchronization enhancement and reached the maximum during seizures [20]. Although brain network research on epilepsy is still at the exploratory stage, epilepsy is a network-level disease that has received increasing recognition, and relevant results have shown that examining epilepsy from a holistic, network and integrated perspective should offer new insight.

Although there have been studies showing that ultrasound neuromodulation has a positive effect on epilepsy, no related research has examined whether and how ultrasound changes epileptic brain networks. The effect of LIFU on epileptic brain connectivity and the synchronization of different brain regions during seizures is of great significance to the exploration of the ultrasound neuromodulation mechanism. In this study, LIFU targeted most of the hippocampus globally rather than its specific subdivisions, so we used intraperitoneal injection of kainic acid (KA). KA is a potent neuroexcitatory and neurotoxic analogue of glutamate. Previous studies have shown that intraperitoneal administration of KA can induce tonic-clonic seizure and limbic motor signs in rats, including wet dog shakes, facial myoclonia and paw tremor. These seizure activities is due to damage to neurons, especially in the hippocampus and amygdaloid complex [21]. We made a multichannel skull surface electrode that could cover the whole brain to record brain activity. We explored stimulation-dependent functional connections derived from EEG data by combining graph theory of network topology with the phase synchronization of electrode interactions. The phase-locking value (PLV) algorithm was used to construct the brain networks, generate the corresponding adjacency matrices, and further calculate the brain network indicators.

## 2. Materials and Methods

### 2.1. Animal Preparation and Grouping

Male Sprague–Dawley rats (300 ± 50 g prior to epilepsy induction; *n* = 21) were housed under a 12-h light/dark cycle and provided food and water. Animal care and handling were conducted according to the guidelines approved by the Institutional Animal Care and Use Committee of the Beijing Institute of Technology. All procedures were carried out in full accord with the Helsinki Declaration on Animal Rights and the Guide for the Care and Use of Laboratory Animals of the National Institutes of Health (NIH publication, 8th Edition, revised 2011 [22]). The procedures in the study were designed to minimize the pain or discomfort of the animals, in accordance with the current protocols approved by the Laboratory Animal Ethics Committee of Beijing Institute of Technology (Beijing, China). Rats were randomly divided into three groups. The rats in Group 1 (*n* = 7) were treated with LIFU sonication after intraperitoneal injection of KA, recorded as ‘KA (+)/LIFU (+)’. The rats in Group 2 (*n* = 7) only received KA without LIFU and were recorded as ‘KA (+)/LIFU (−)’. Group 2 was used as a control variable for comparison with Group 1. The rats in Group 3 (*n* = 7) only received LIFU without epileptic induction, recorded as ‘KA (−)/LIFU (+)’. After the experiment, the rats were euthanized using intraperitoneal injections of pentobarbital (150 mg/kg).

### 2.2. Computational Simulation Modeling and Ultrasonic Field Distribution

To understand the spatial distribution of ultrasound-induced pressure waves in the brain, we constructed a simple finite element method (FEM) model by COMSOL Mutiphysics software (COMSOL AB, Stockholm, Sweden). The modeling domain consists of an elliptical cylinder (d1 (major axis) = 16 mm, d2 (minor axis) = 10 mm, h = 20 mm), which approximates brain tissue and was surrounded by a 1 mm thick ring representing the skull. This simple 3D geometry grid-measurement approximation of the rat head as a cylinder offers a reference value to understand the basic transmission behavior of ultrasound [23]. The density (ρ) of the skull was designed as 1912 kg/m^3^, and the speed of ultrasound (c) was estimated to be 2300 m/s [24]. For the brain tissue, ρ was set to 1030 kg/m^3^, and c was designed as 1550 m/s [25]. The ultrasonic gel in the collimator was configured to have the same material properties as water. Changes in the acoustic intensity (I_SPTA_), mechanical index (MI) and sound pressure with depth in the brain were calculated and plotted.

### 2.3. Electrode Positioning and Implantation

**Figure 1:**
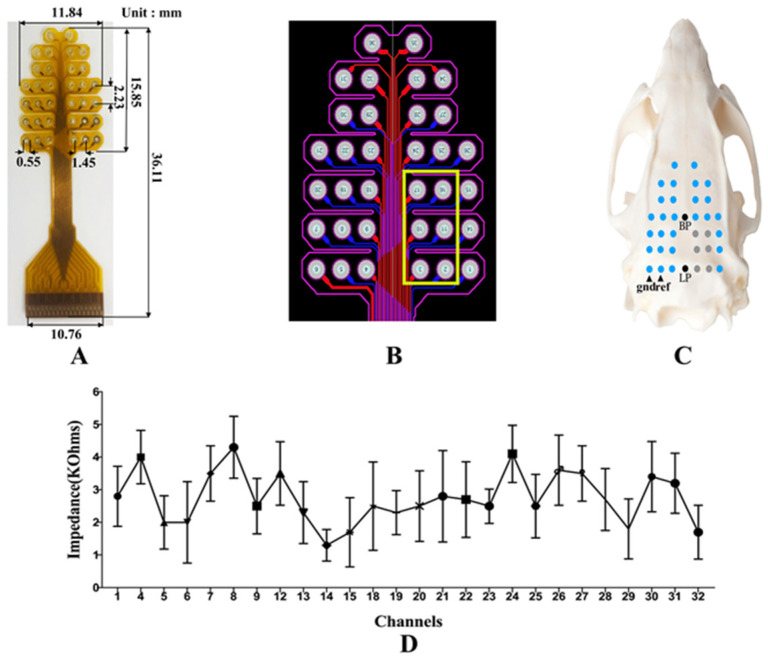
(**A**) Self-developed 32-channal EEG electrode and its size. (**B**) The electrode circuit diagram, the part inside the yellow box is the position reserved for the ultrasound transducer. (**C**) The relative position of the electrode on the skull surface. (**D**) Impedance of each channel of the electrode.

**Figure 2:**
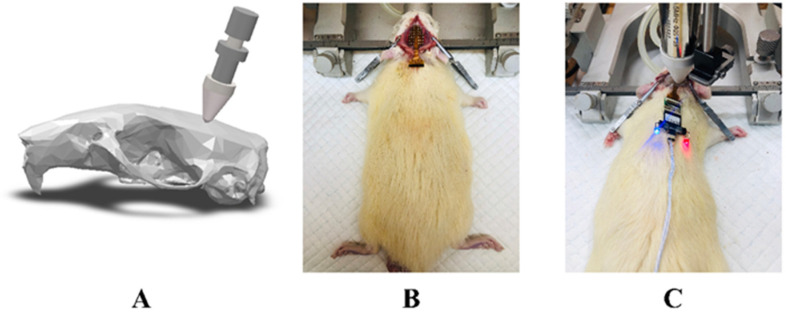
(**A**) Schematic diagram of ultrasound transducer and skull model. (**B**) Implantation with an EEG electrode. (**C**) A rat undergoing sonication.

A self-developed 32-channel EEG electrode was used to record EEG signals as shown in Figure 1A. The electrode has been used in previous EEG experiments and related article has been published, and its safety and effectiveness have been confirmed [26]. The size of the electrode and the distance between the channels are shown in Figure 1A. The assembly of the electrode array on rat skull is shown in Figure 1C, the center point of the last row was at the Lambda point, and the center point of the fourth row was at the Bregma point. The skull nails penetrated the rat skull to reach the epidural space without damaging the dura mater. In order to prevent signal distortions impedances at each electrode contact with the scalp should all be bellow 5K Ohms [27]. We checked the electrode impedance by an external equipment nanoZ (Plexon OmniPlex, Hong Kong Plexon Co., Ltd., Hong Kong, China) before recording. The results of multiple tests are shown in Figure 1D. To have enough space to place the ultrasound transducer on the skull and avoid the chaos effect caused by the superposition of ultrasound and EEG, the 2nd, 3rd, 10th, 11th, 16th, and 17th channels were artificially vacated, as shown in Figure 1B. All surgical procedures were performed using sterile techniques under full general anesthesia with 1.5% isoflurane in oxygen-enriched air. A gas evacuation apparatus (R546W, RWD Life Science Co., Ltd., Shenzhen, China) was used to maintain anesthesia throughout the whole procedure without interruption. Surgery included exposing the skull and cleaning the skull surface (using 2.5–3.5% hydrogen peroxide). We surgically peeled the scalp of rats and created holes in the skull to attach the electrodes to the skull surface through the skull nails without electrically-conductive gel as shown in Figure 2B, improving stability and reliability. Compared with craniotomy, this approach greatly reduces the damage to animals, improving the stability and reliability of the method. Before the implantation operation, penicillin solution was injected intramuscularly to prevent inflammation, and after the suture, penicillin solution was used to clean the incision. After the electrode was fixed, a special adapter was used to connect the electrode to the signal acquisition device (Plexon OmniPlex, Hong Kong Plexon Co., Ltd., Hong Kong, China). The rat was fixed on the stereotaxic coordinate apparatus (E03135-001, RWD Life Science Co., Ltd., Shenzhen, China) with ear bars and a clamping device. A gas evacuation apparatus (R546W, RWD Life Science Co., Ltd., Shenzhen, China) was used to maintain anesthesia throughout the whole procedure without interruption, and a consistent anesthesia level was maintained. A temperature controller (Serial No. C4L02-010, RWD Life Science Co., Ltd., Shenzhen, China) was used to maintain proper body temperature at ~37 °C. One week after the surgery, the ultrasound stimulation was performed if the recovery of the rats was good.

### 2.4. LIFU Sonication and Induction of Epilepsy

**Figure 3:**
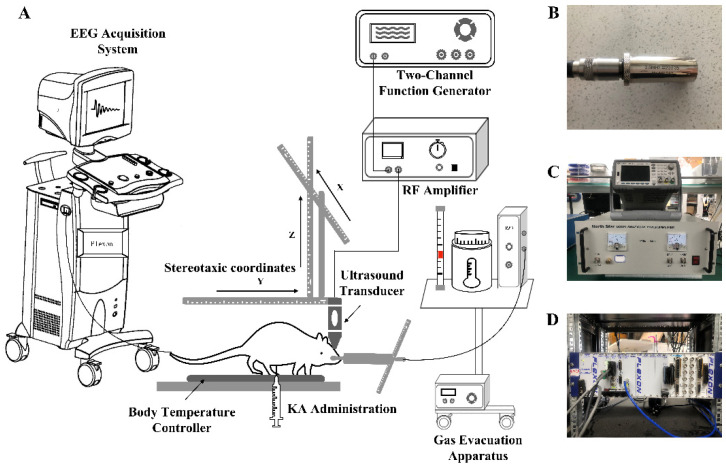
Basic ultrasonic brain stimulation rig. (**A**) Schematic view of the experimental apparatus. (**B**) Shown is a focused ultrasound transducer with a 0.5 MHz center frequency. (**C**) Top, a two-channel waveform generator used to trigger ultrasound pulses. Bottom, an RF amplifier used to receive an input voltage waveform to provide the output power to the transducer for producing the acoustic pressure profile of an LIFU stimulus waveform. (**D**) Shown is the signal acquisition device.

**Figure 4:**
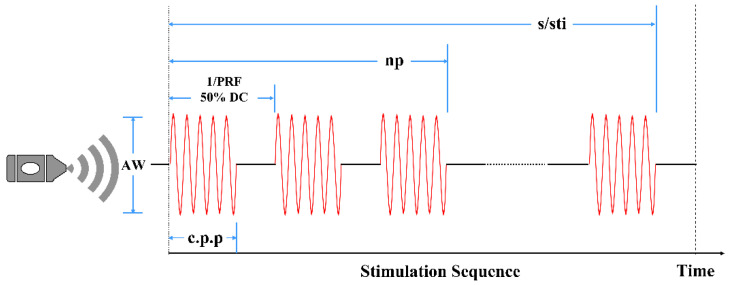
Pulsed ultrasound waveform generation with the following properties: Af = 0.5 MHz, c.p.p. = 150, PRF = 1.5 kHz, np = 200.

In animals, the optimal waveforms between transcranial transmission and brain absorption for evoking intact brain circuit activity have been reported to be composed of acoustic frequencies ranging between 0.25 and 0.65 MHz according to both mathematical models and experimental data. On this basis, a focused ultrasound transducer with a 0.5 MHz center frequency (35 mm focal depth, 20 mm in diameter; Goworld, Guangdong, China) was used. The driving signal was derived from a two-channel waveform generator (33500B, Keysight Technologies Inc., Santa Rosa, CA, USA) and amplified through a radio frequency amplifier (North Star model SWA200D RF power amplifier, the Institute of Acoustics of the Chinese Academy of Sciences, Beijing, China). The achievement of the experimental platform was published at the ICCIIBMS conference [28]. Figure 3 is a schematic view of the experimental apparatus. An example of the LIFU waveform is shown in Figure 4. The ultrasound transducer was fixed over the rat and connected to the rat skull by a 3D printed conical acoustic collimator filled with US gel, as shown in Figure 2C.

**Figure 5:**
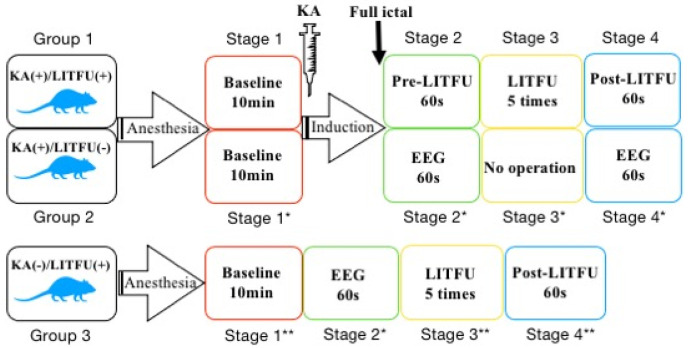
Experimental flow chart. Stage 1 (Stage 1*/1**) represents the baseline period. The baseline EEG was recorded for ten minutes after the EEG signals stabilized following the administration of anesthesia. Stage 2/2* (named as ‘Pre-LIFU’) in Group 1 and Group 2 indicate the time-interval after observing significant evidence of ictal behavior (e.g., forelimb clonus and tail-twitches) and just before the sonication. Stage 3/3** in Group 1 and Group 3 represent the 5 times of the sonication (named as ‘LIFU’). There was no operation in Stage 3 * of Group 2. Stage 4/4** represents the time-interval after the sonication. Stage 4 * in Group 2 represents the EEG data corresponding to the time of the other two groups.

After the experiment started, the rats were anesthetized under full general anesthesia with 1.5% isoflurane in oxygen-enriched air first, and the EEG signals were recorded at a 1000 Hz sampling rate. In order to clarify the operation process, we divided the entire experiment into four stages. Baseline data were recorded for 10 min (shown as ‘Stage 1′, ‘Stage 1 *’ and ‘Stage 1 **’ in Figure 5). KA solution (6.5 mg/kg, based on animal weight) (2 mg/1 mL in 0.9% saline, No. k0250-10MG, Sigma-Aldrich, St Louis, MO, USA) was administered to the rats in Group 1 and Group 2 via intraperitoneal injection. In the preliminary experiment, five rats were injected with this dose of KA, and all rats showed obvious seizure behavior (e.g., forelimb clonus and tail-twitches) after 20–30 min under anesthesia, and the seizure lasted for more than 2 h. In the experiment, we judged the seizures of each rat by forelimb clonus and tail-twitches. After the epilepsy behavior manifested in the rats, EEG data were continuously recorded in Group 2. When seizures lasted 60 s in Group 1, sonication was then applied 5 times (noted as ‘Stage 3′ in Figure 5). After sonication, EEG data were recorded for 60 s (noted as ‘Stage 4′ Figure 5). After all operations were completed, the epileptic seizures were terminated by intraperitoneal injection of diazepam (10 mg/kg). The rats in Group 3 only received sonication without epileptic induction. The experimental flow is shown as Figure 5.

### 2.5. Brain Network Construction and Graph Theoretical Analysis

The EEG signals were preprocessed with EEGLAB (Swartz Center for Computational Neuroscience, La Jolla, CA, USA). A 50 Hz notch filter was used to remove power frequency noise, and a bandpass filter with a cutoff frequency of 0.1 Hz to 48 Hz was used to remove high-frequency signals. To improve calculation speed, the sampling rate was reduced to 250 Hz. Broad (0.1~48 Hz), delta (0.1~4 Hz), theta (4~8 Hz), alpha (8~12 Hz), beta (12~30 Hz), and gamma (30~48 Hz) bands were extracted in sequence from the preprocessed signals, and we constructed the brain networks of these six frequency bands. We used the PLV to construct the neuronal network to calculate the indicators of the brain network. This method calculates the instantaneous phase of a time-varying signal and statistically analyzes it within a given time period to characterize the phase change relationship of the two signals. Its advantage is that the influence of signal amplitude can be eliminated, and the synchronization of two time series (such as two EEG channels) can be observed from the phase. We used HERMES (Centre for Biomedical Technology, Technical University of Madrid, Madrid, Spain) to calculate adjacency matrices. Each channel of the electrode served as a node of the network, and the relevant PLV value calculated was defined as the edge of the network.

**Figure 6:**
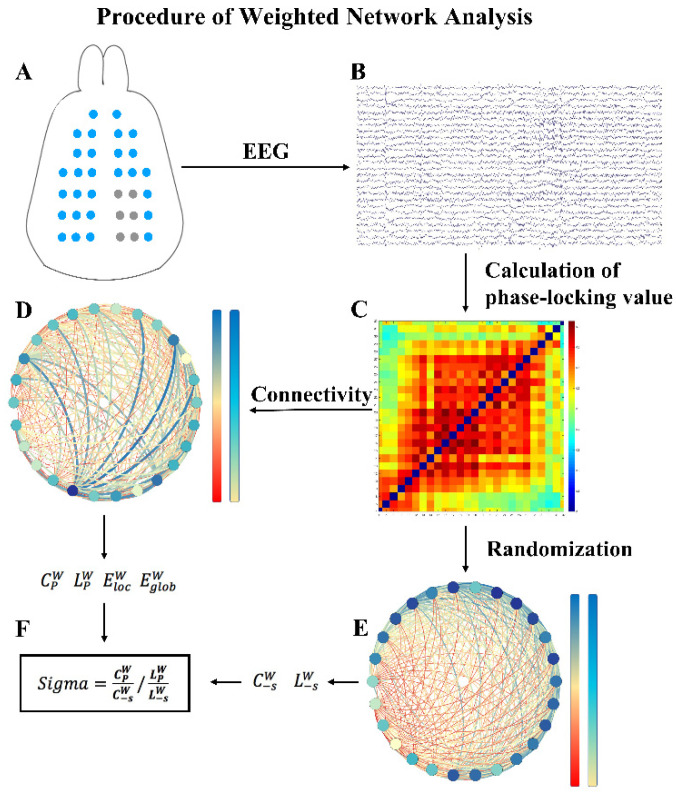
Program diagram of the procedure involved in weighted networks analysis of EEG recordings. (**A**) EEG signals were recorded from electrodes. (**B**) The acquired EEG signals were filtered within five frequency bands. (**C**) The PLV was estimated as a measure of correlation between all pairs of channels, resulting in correlation matrices. (**D**) Then, weighted networks were constructed based on the correlation matrices. The clustering coefficient $C_{P}^{W}$, characteristic path length $L_{P}^{W}$, global efficiency $E_{glob}^{W}$ and local efficiency $E_{loc}^{W}$ were calculated to characterize each graph. (**E**) In addition, surrogate graphs were derived by randomly shuffling the cells of the correlation matrices. (**F**) For each original graph, network indicators were assessed and averaged over the ensemble of surrogate graphs, resulting in $C_{-s}^{W}$ and $L_{-s}^{W}$. Finally, the small-worldness $Sigma=\frac{C_{P}^{W}}{C_{-s}^{W}}/\frac{L_{P}^{W}}{L_{-s}^{W}}$ was determined.

We selected a data length of 60 s to calculate adjacency matrices. We calculated indicators by GRETNA (State Key Laboratory of Cognitive Neuroscience and Learning, Beijing Normal University, Beijing, China). As global statistical characteristics of a functional brain network, we calculated brain network indicators (including the path length aLp, clustering coefficient aEg, small-worldness aSigma, local efficiency aEg and global efficiency aEloc). Table 1 provides detailed descriptions of the above indicators. In addition, the areas under the curve (AUCs) of indicators were calculated for each network measurement to provide a scalar that did not rely on the given threshold selection. The flow of data processing is shown in Figure 6. Most previous studies have focused on binary networks because of the reduction in computational complicacy and clarity of network metric definitions. However, binary networks ignore the strength of connections below the threshold and consequently fail to identify subtle network structure. Therefore, we did not binarize the brain networks but used the weighted networks for analysis.

SPSS version 26.0 (SPSS, Inc., Chicago, IL, USA) was used for statistical analyses. The statistical results are expressed as the mean ± standard error of the mean (SEM). In the between-group analysis, the Mann–Whitney U test was performed separately between the corresponding stages in the two groups to examine the effects of LIFU. In the within-group analysis, the Wilcoxon matched pairs test was used to detect differences in synchronization and other brain network indicators between Stage 2 and Stage 4 (Stage 2* and Stage 4*). All statistical comparisons were two-tailed with α = 0.05.

## 3. Results

### 3.1. Simulated Sound Field Distribution and Line Graph

**Figure 7:**
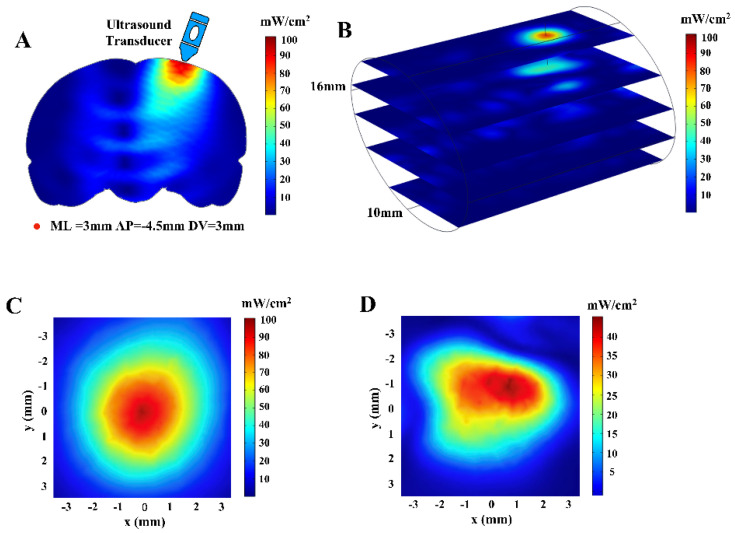
Finite element modeling and ultrasonic intensity (I_SPTA_) distribution of the brain. (**A**) Schematic diagram of the target encephalic region stimulated by ultrasound. (**B**) Three-dimensional distribution of sound intensity in the finite element model. (**C**) The two-dimensional ultrasound distribution in the xy plane with the highest ultrasonic intensity in the brain. (**D**) The two-dimensional ultrasound distribution in the xy plane at DV = 3 mm.

**Figure 8:**
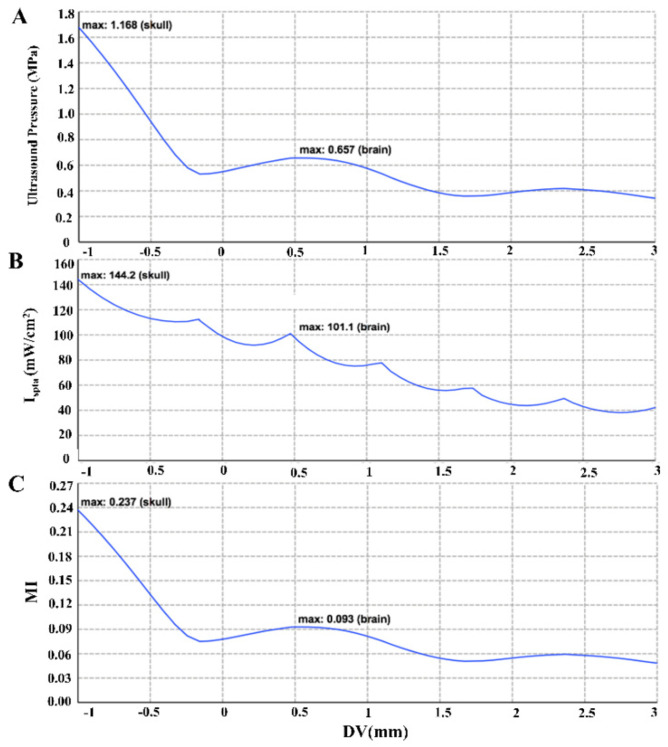
(**A**) The change in sound pressure as the depth in the brain increases. (**B**) The change in sound intensity as the depth in the brain increases. (**C**) The change in mechanical index with increasing brain depth. Point zero on the x axis represents the boundary between the brain tissue and the skull, the part less than zero (−1–0 mm) represents the skull, and the part greater than zero (0–4 mm) represents the brain tissue.

The FEM model we constructed reflected the distribution of the sound field in the brain, as shown in Figure 7. The ultrasound waves could be transmitted to the hippocampus of the rat brain by placing the ultrasound transducer at the position set in this paper. Figure 8 shows the maximum sound pressure, the maximum I_SPTA_ and the maximum MI in different depth planes with changes in brain depth. The maximum sound intensity of sonication used in this study was 101.1 mW/cm^2^ (I_SPTA_) which was far below the upper regulatory limit of safety stipulated by American Institute of Ultrasound in Medicine (AIUM; 720 mW/cm^2^; [29]). The MI of this study was 0.093 which was adequately within the range of safety guidelines (i.e., 0.23; [29]).

### 3.2. Epileptic Raw EEG Analysis

**Figure 9:**
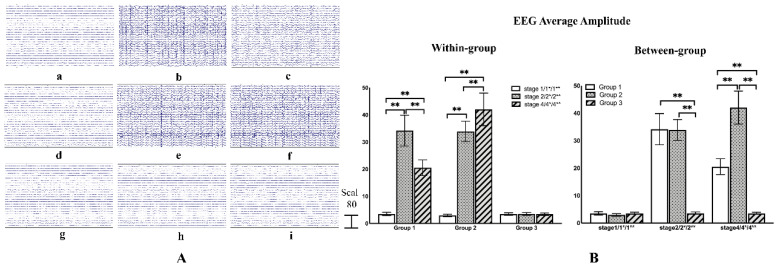
(**A**) (**a**–**c**) Respectively represent the raw EEG data of Stage 1, Stage 2, and Stage 4 in Group 1. (**d**–**f**) Respectively represent the raw EEG data of Stage 1*, Stage 2*, and Stage 4* in Group 2. (**g**–**i**) Respectively represent the raw EEG data of Stage 1 **, Stage 3**, and Stage 4** in Group 3. (**B**) Within-group comparisons and between-group comparisons of EEG average amplitude. ** indicates significance (*p* < 0.01).

In the experiment, all rats in Group 1 and Group 2 were successfully induced epilepsy. We judged epileptic seizures by observing forelimb clonus and tail-twitches. According to the Racine scoring system, when the rat showed forelimb clonus and tail-twitches, it was considered full ictal activity [30]. Figure 9A shows the raw EEG at different stages of one rat selected in each group. The EEG potential of Group 1 and Group 2 both showed obvious changes after the epileptic induction, while the EEG potential of Group 1 was weakened after LIFU, however, there was no obvious change in Group 2. In Group 3 there was no significant change in all three stages and the acoustic startle reflex was not found. The raw EEG amplitude of each rat was superimposed and averaged, and one way ANOVA-test was used for between-group and within-group analysis. The result has been shown in Figure 9B. It can be seen that in the within-group analysis, the average amplitude in Group 1 increased significantly after the epileptic seizure (*p* < 0.01, F (1,2) = 86.410), while the average amplitude decreased significantly after sonication (*p* < 0.01, F (1,2) = 86.410). However, compared to Stage 2*, the average amplitude of Group 2 continued to rise in Stage 4* (*p* < 0.01, F (1,2) = 125.790). In the between-group analysis, there is no significant difference between Group 1 and Group 2 in the two stages before sonication. After sonication, the EEG average amplitude in Group 1 was significantly lower than Group 2 (*p* < 0.01, F (1,2) = 101.188). There was no significant change in all three stages in Group 3. We then analyzed the changes in brain connectivity to explore the deeper reasons.

### 3.3. Phase-Locking Value Changes before and after Sonication

**Figure 10:**
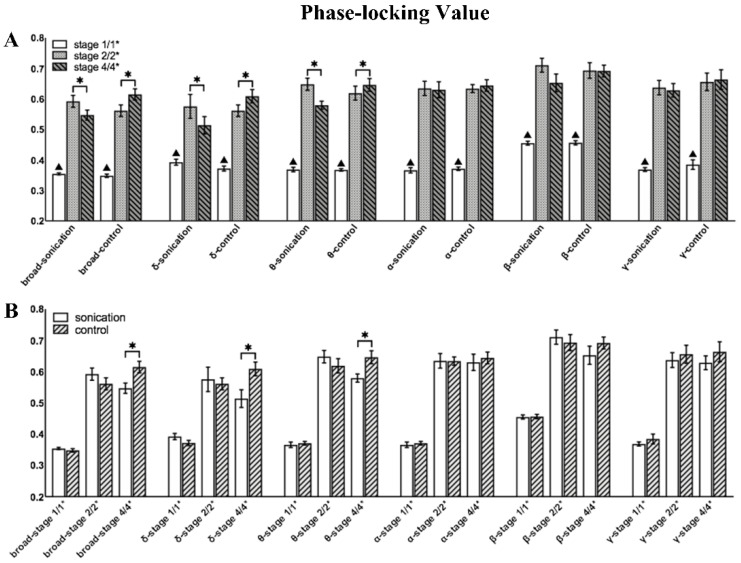
Mean phase-locking value (PLV) for all filtered frequency bands (delta 0.1–4 Hz, theta 4–8 Hz, alpha 8–13 Hz, beta 13–30 Hz and gamma 30–48 Hz) and the broad-filtered signal (0.1–48 Hz). (**A**) Within-group comparisons of the mean PLV at the different stages; ▲ represents a significant difference between Stage 1/1* and Stage 2/2* (*p* < 0.05). (**B**) Between-group comparisons of the mean PLV between Group 1 and Group 2 (‘Sonication’ represents Group 1 and ‘Control’ represents Group 2). * indicates significance (*p* < 0.05).

**Figure 11:**
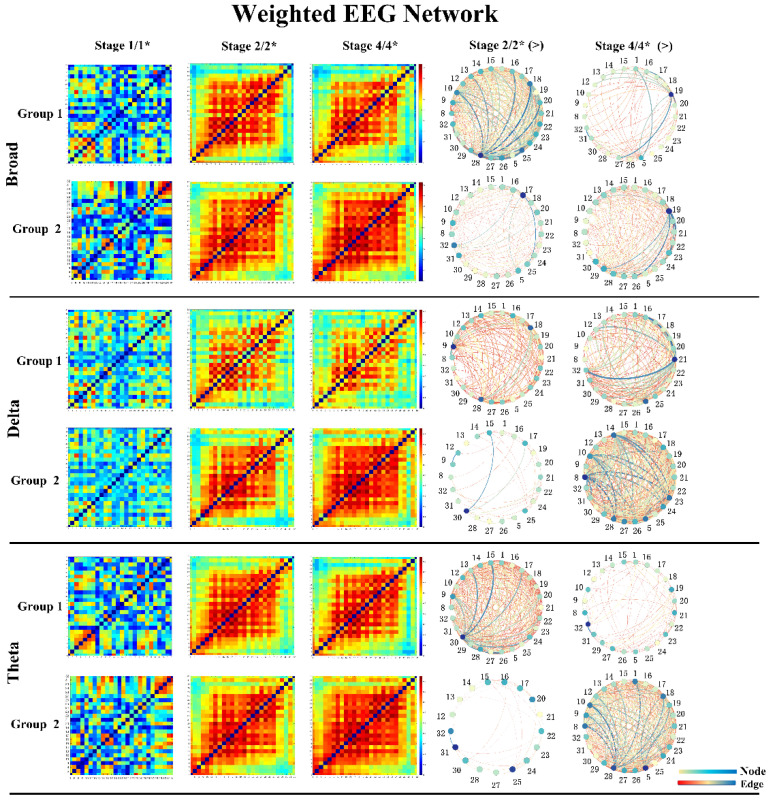
The mean adjacency matrices of three different stages of the broad, delta, and theta frequency bands in the two groups. In addition, there are different connections between the two stages in each group, Stage 2/2* and Stage 4/4*. The first is that the connections of Stage 2/2* before sonication are greater than those of Stage 4/4*, and the latter is that the connections of Stage 4/4* after sonication are greater than those of Stage 2/2*.

**Figure 12:**
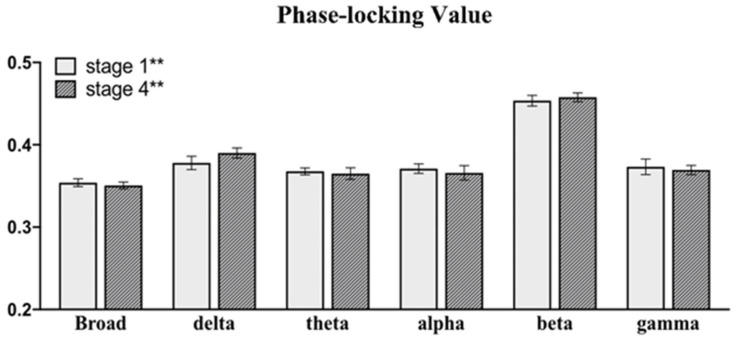
Mean phase-locking value (PLV) for all filtered frequency bands (delta 0.1–4 Hz, theta 4–8 Hz, alpha 8–13 Hz, beta 13–30 Hz and gamma 30–48 Hz) and the broad-filtered signal (0.1–48 Hz) in Group 3.

As shown in Figure 10, the PLV value of each frequency band significantly increased after a seizure. In the within-group analysis of Group 1, the PLV values of the broad, delta and theta bands decreased significantly. In Group 2, the PLV of the delta, theta and broad bands increased significantly, and no significant difference was found in other bands. In the between-group analysis, the PLV of Stage 1/1* and Stage 2/2* showed no significant difference in all bands, which confirmed that the degree of seizure was similar in the stages before sonication. In the broad delta and theta bands, the PLV of Group 1 was significantly lower than that of Group 2 in Stage 4/4*. Figure 11 shows the changes in the mean PLV of the three frequency bands with significant differences at three different stages and the comparison of the brain network connections before and after sonication in Group 1. As shown in Figure 12, there was no significant difference in the PLV of each frequency band in the two stages before and after LIFU in Group 3.

### 3.4. Brain Network Indicators before and after Sonication

**Figure 13:**
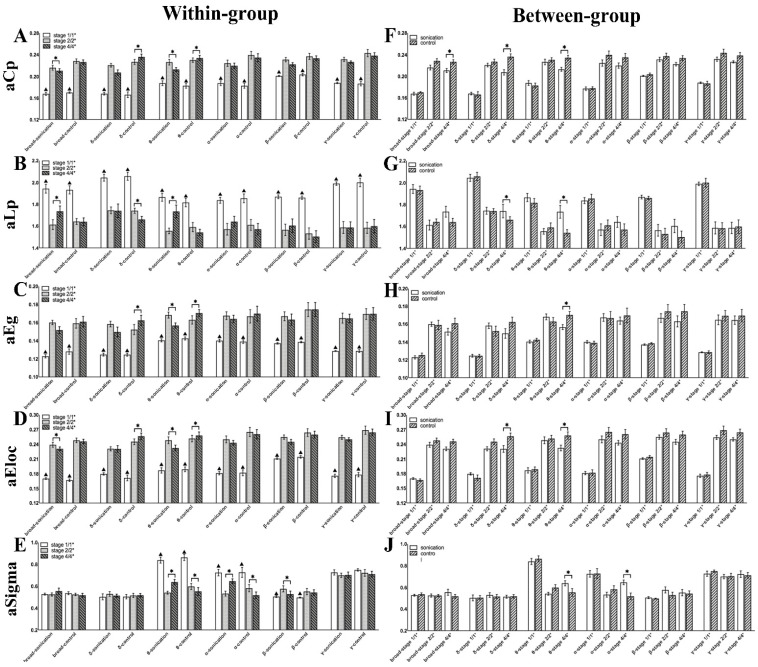
Brain network indicators (aCp, aLp, aEg, aEloc, and aSigma) for all filtered frequency bands (delta 0.1–4 Hz, theta 4–8 Hz, alpha 8–13 Hz, beta 13–30 Hz and gamma 30–48 Hz) and the broad-filtered EEG (0.1–48 Hz). (**A**–**E**) Within-group comparisons of brain network indicators at different stages. (**F**–**J**) Between-group comparisons of brain network indicators between Group 1 and Group 2 (‘Sonication’ represents Group 1 and ‘Control’ represents Group 2). * indicates significance (*p* < 0.05), ▲ represents represents a significant difference between Stage 1/1* and Stage 2/2* (*p* < 0.05).

As shown in Figure 13, in within-group analysis, the four indicators except aSigma had significant changes in all frequency bands after seizure (*p* < 0.05). aCp decreased significantly in the broad and theta bands after sonication. There was also a downward trend in other frequency bands but no significant difference. In Group 2, aCp increased significantly in the delta and theta bands during the normal course of a seizure. aLp showed an upward trend after sonication and showed a significant difference in the broad and theta bands, while in Group 2, it showed a significant downward trend in the delta band. Global efficiency aEg showed a significant downward trend in the theta band after sonication, while in Group 2, it showed a significant increase in the delta and theta bands. Local efficiency aEloc decreased significantly in the broad and theta bands after sonication, while in Group 2, aEloc of the delta and theta bands increased significantly during the normal course of a seizure. In broad, delta, gamma, and beta bands, aSigma of the three stages showed no significant differences. After sonication, aSigma showed a significant increase in both theta and alpha bands. In Group 1, there was a significant decrease in both theta and alpha bands. It is worth noting that the beta band presented a completely different trend from the theta and alpha bands. After seizure, aSigma increased significantly, but after sonication, there was a significant downward trend in Group 1. Table 2 shows the p values in the between-group comparison.

In the between-group analysis, all the indicators of Stage 1/1* and Stage 2/2* between Group 1 and Group 2 did not show significant differences in each frequency band, which proved that the two groups of rats had basically the same degree of seizure before sonication. aCp of Group 1 was significantly lower than that of Group 2 in the broad, delta and theta bands. aLp of the delta and theta bands in Group 1 was significantly higher than that in Group 2 after sonication, and there were similar trends in other frequency bands, but there was no significant difference. aEg in Group 1 was significantly lower than Group 2 in the theta band, and aEloc was significantly lower than Group 2 in the delta and theta bands. The small-world aSigma in Group 1 was significantly higher than that in Group 2 in the theta and alpha bands. Table 3 shows the p values in the between-group comparison.

## 4. Discussion

Although the physiological significance of EEG signals in different frequency bands is still indistinct, the oscillating activities of different frequency bands play different roles in understanding the local and global functional integration of the brain. Our results indicated that LIFU sonication reduced the PLV during epileptic seizures, that is, it inhibited the strength of the epileptic brain network connections. Several important brain network indicators were also changed: the weighted clustering coefficient decreased, the weighted characteristic path length increased, and the weighted global efficiency and local efficiency decreased. Moreover, the small-worldness aSigma increased, especially in the low-frequency bands below 12 Hz. In Group 2 without sonication, the strength of the brain network connections and the brain network indicators did not change, and there was even an opposite trend.

Ordered networks have a high clustering coefficient and a long characteristic path length, which are conducive to the local information transmission of the network; random networks have a low clustering coefficient and a short characteristic path length, which are conducive to the global information transmission of the network. Watts and Strogatz proposed the concept of a small-world network in 1998 [31]. Small-world networks not only have a high clustering characteristic similar to ordered networks but also a low characteristic path length similar to random networks. This is the most effective network topology structure that configures and optimizes the transmission of information. This topological structure can ensure that the brain consumes the smallest amount of resources to complete the largest function and achieve the optimal connectivity so that the brain network can complete more complex functions with fewer connections. At present, brain graph theory analysis based on EEG, fMRI, and MEG data all showed small-world characteristics [32,33,34,35].

The PLV evaluates the strength of the brain network connections; the higher the strength of the connection, the higher the synchronization among brain regions. From our results, it is clear that the PLV of theta and broad bands after LIFU sonication were significantly reduced within and between groups, while the PLV of delta bands in Group 2 were significantly increased. It showed that in the normal process of seizures, the PLV gradually increased in the delta band and did not decrease in other frequency bands. This result indicated that in the early stage of a seizure episode, the brain network connections became stronger, and the synchronization among the various brain regions increased, which was conducive to the transmission of epileptic signals in the whole brain. LIFU stimulation reduced the connection strength of the brain network in the theta band.

The clustering coefficient evaluates the local information transmission capability of the network. The higher the clustering coefficient is, the stronger the local transmission capability is, and correspondingly, the greater the local efficiency of the network is. In one group, the clustering coefficient and local efficiency in the broad and theta bands decreased significantly after sonication, while in Group 2, the clustering coefficient and local efficiency in the delta and theta bands increased significantly, while in the other bands, there was no obvious change. Therefore, the trends of aLp and aEg were consistent with each other. This meant that in the course of a normal epileptic seizure, the local transmission capacity of brain networks in the early epileptic stage significantly increased. The characteristic path length evaluates the global information transmission capacity of networks. The smaller the characteristic path length is, the stronger the global transmission capacity is, and accordingly, the greater the global efficiency of networks is. In Group 1, the characteristic path length of the broad and theta bands increased significantly; however, in Group 2, the characteristic path length of the delta band decreased significantly, and the other frequency bands did not change significantly. The trends of aCp and aEloc were also consistent with each other. This meant that in a normal epileptic seizure episode, the global transmission capacity between brain networks was increasing. The increase in global efficiency and local efficiency was conducive to the transmission of epileptic signals in the whole brain and the local brain region and allowed a high degree of synchronization among various brain regions. LIFU sonication inhibited transmission and hindered the development of a high degree of synchronization among various brain regions, especially in the low-frequency bands. This may mean that LIFU suppresses epileptic signal transmission by adjusting the clustering coefficient and characteristic path length of the delta and theta bands, thereby suppressing epileptic seizures.

The small-worldness aSigma of the theta and alpha bands decreased significantly after LIFU sonication. In the between-group analysis, no significant difference was found in Stage 2/2* of the two groups, while aSigma of the theta and alpha bands in Group 1 after sonication was significantly lower than that in Group 2. The results showed that during normal epileptic seizures, aSigma of the theta and alpha bands decreased significantly after the seizure episode and then continued to decrease gradually with the development of epilepsy. After sonication, aSigma of these two frequency bands increased; although it did not recover to the level before the seizure, it tended to normalize. Netoff and Chavez found that a random network even had a stronger tendency to synchronize, which suggests that the random interictal neuronal network configuration causes seizures [36,37], which was consistent with our results. It provided a basis for us to further analyze the important role of the brain network in the mechanism of ultrasound neuromodulation. It is worth noting that the changes in the beta band were diametrically opposed to theta and alpha band activity, which may be a compensation mechanism in the brain.

The hippocampus is an important brain region in the limbic system that controls behavior and physiological functions. KA mainly causes abnormal neuronal hyperexcitability and histopathological lesions in the bilateral hippocampus of the brain, similar to human temporal lobe epilepsy [38,39]. The theta band is the dominant frequency of the hippocampus, and changes in theta band have been widely confirmed during the development of epilepsy. Douw, et al. reported that increased synchronization of the theta band was shown in patients with different types of epilepsy [40]. Laetitia, et al. believed that the changes in the hippocampal circuit caused by the original injury affected the normal theta rhythm mechanism. In view of this information, it was speculated that hippocampal damage affected other brain regions through changes in the theta band and eventually resulted in a seizure. This may mean that low-intensity pulsed ultrasound inhibits epilepsy by adjusting the strength of the brain network connections in the theta band.

Gavrilov, et al. found that the main effect of ultrasound in stimulating neural structures is due to mechanical force that could produce alterations in membrane potential that could stimulate the nervous system, and it has also been proposed that ultrasonic sonication may influence membrane fluidity, turbidity and permeability [41,42,43]. Since seizure activity is caused by abnormally excessive or synchronous neural activity in the brain [44], and synaptic contacts could potentially be disrupted by ultrasound waves [45], LIFU sonication might reduce the transmission of epileptic discharges across the brain. Although little is understood about the detailed mechanism underlying ultrasound neuromodulation, we conjectured that ultrasound suppressed epileptic signal transmission by affecting the brain network connections of the theta band and finally inhibited epileptic seizures. It can be hypothesized that ultrasound controls neural circuits and the central nervous system by affecting brain functional connections, especially the low-frequency band below 12 Hz, as the main frequency band of the hippocampus. This can also explain the significant changes in the indicators of the brain network connections before and after ultrasonic sonication.

It has long been reported that ultrasound can significantly affect the neurophysiology of in vitro local neural circuitry. Gavrilov, et al. reported that the main effect of ultrasound in stimulating neural structures is due to mechanical force that could produce alterations in membrane potential that could stimulate the nervous system, and it has also been proposed that ultrasonic sonication may influence membrane fluidity, turbidity and permeability. Accordingly, the activity of ion channels or receptors on the membrane can be influenced by ultrasound sonication, and the transmembrane concentrations, passage of ions or passage of neurotransmitters can be subsequently altered. Although the mechanism of ultrasound neuromodulation is still unclear, our results present a new hypothesis for the mechanism of ultrasound neuromodulation. We conjectured that ultrasound suppressed epileptic signal transmission by affecting the brain network connections of the theta band and finally inhibited epileptic seizures. It can be hypothesized that ultrasound controls neural circuits and the central nervous system by affecting brain functional connections, especially the low-frequency band below 12 Hz, as the main frequency band of the hippocampus. This can also explain the significant changes in the indicators of the brain network connections before and after ultrasonic sonication.

Recently, Qi, et al. founded that ultrasound stimulation can elicit the inward current and action potentials in cultured auditory cortical neurons in vitro [46]. In addition, previous studies shown that ultrasound can induce motor movements and elicit an auditory startle response in lightly anesthetized animals [47,48]. However, King, et al. used 500 KHz ultrasound to induce somatomotor response of rodents in varying levels of anesthesia (0.05%, 0.1%, 0.5% iso), they reported that the behavioral responses were not a startle response to auditory stimuli though a controlled experiment [49]. The rats in Group 3 of our study only received LIFU without epileptic induction in the same level of anesthesia (1.5% iso), and the startle response was not found. Although there was no startle response and audible high-pitched noise in our study, ultrasonic neuromodulation can cause indirectly auditory activation [50]. In our study, LIFU focused on the hippocampus after passing through the secondary visual cortex. The cortical stimulation may partially explanatory to our results, because extensive connections and interactions exist among different brain circuits [51,52,53]. Although LIFU successfully reduced the epileptic network connections in our study, as to how much the contribution of different cortex pathway needs further investigation.

Considering biosecurity, it is worth noting that ultrasonic sonication can potentially generate free radicals. These free radicals, although short-lived, are extremely unstable and can react easily with other surrounding biological molecules, possibly causing tissue damage and inflammation [29]. However, these free radicals are typically produced at high acoustic intensities that are associated with cavitation [54]. Tissue damage has not been caused in studies implementing nonthermal bioeffects of ultrasound to modulate neuronal activity [2,49,55,56,57,58]. Because the present study used an acoustic intensity much lower than those that produce cavitation and free radicals, sonication in the present study was unlikely to have a negative impact on brain tissue. The maximum sound intensity of sonication used in this study was 101.1 mW/cm^2^ (I_SPTA_), which was far below the upper regulatory safety limit stipulated by the American Institute of Ultrasound in Medicine (AIUM; 720 mW/cm^2^; [59]). The MI of this study was 0.093, which was adequately within the range stated in the safety guidelines (i.e., 0.23; [59]). Yu, et al. used 0.5 MHz focused ultrasound to investigate the effect of ultrasound stimulation on different functional neuron types in anesthetized rodent brains. Hematoxylin and eosin stains gathered immediately after stimulation at S1 cortices showed no neuronal damage, local hemorrhage or inflammatory response at the stimulation site [60]. Tufail, et al. successively examined how low-intensity ultrasound influenced blood-barrier, probed the cellular-level consequences of ultrasound on brain tissues, used quantitative transmission electron microscopy to determine the effects of ultrasound on brain ultrastructure, examined if transcranial ultrasound stimulation of motor cortex produced any gross impairments in motor behavior. In all of the above tests, any neurological abnormalities such as paralysis, ataxia, or tremor have never been observed in these rodents [2]. In conclusion, the ultrasonic parameters we used were all within the range of safety guidelines for clinical ultrasound. The experimental results of Group 3 also show that LIFU has no effect on the EEG and the PLV value of each frequency band, and also prove that LIFU has no effect on the normal EEG and brain connectivity.

## 5. Conclusions

The current study still has some technical limitations to address. To research the effectiveness of LIFU in suppressing region-specific epileptogenic activity, a regional chemical kindling model such as an intracortical KA injection in the brain can be used to induce focal epileptic lesions. In addition, we should further study on the efficacy of the parameters and the different effects on brain network connections are needed. It is necessary to choose LIFU parameters carefully to formulate reasonable and detailed treatment guidelines. In future research, we consider extending the experimental period and take necessary technical measures to monitor the behavior of animals. We will add histological examination to further research. The indirect auditory activation and the auditory-related brain network changes of ultrasound neuromodulation will also be further investigated. From our research results, it can be concluded that LIFU-mediated regional specific functional neuromodulation is expected to become a powerful method for studying brain function and neurological diseases, and the influence of LIFU on brain network connections is likely to provide a new research direction for the mechanism of ultrasound neuromodulation. In future research, we will aim to overcome the aforementioned technical shortcomings and continue to conduct in-depth research on ultrasound neuromodulation.

## Figures and Tables

**Table 1:** Brief descriptions of complex network indicators in this paper.

Indicator	Character	Description
**Weighted clustering coefficient**	$C_{P}^{W}$	The extent of local clustering or cliquishness of a network
**Shorted weighted path length**	$L_{P}^{W}$	The extent of the overall routing efficiency of a network
**Sigma**	σ	The small-worldness indicating the extent of a network regarding randomness and order
**Weighted local efficiency**	$E_{loc}^{W}$	How efficiently information is propagated to the direct neighbors of a node
**Weighted global efficiency**	$E_{glob}^{W}$	How efficiently information is propagated through the whole network

Note that indicators listed here only for weighted network.

**Table 2:** Within-group comparisons of weighted brain network.

*p*-Value	Group 1						Group 2					
Frequency Band	PLV	aCp	aLp	aEg	aEloc	aSigma	PLV	aCp	aLp	aEg	aEloc	aSigma
**0.1–48 Hz**	0.1018	0.018	0.043	-	0.018	-	0.018	-	-	-	-	-
**0.1–4 Hz**	0.028	-	-	-	-	-	0.018	0.018	0.018	0.018	0.028	-
**4–8 Hz**	0.018	0.018	-	0.018	0.018	0.018	0.018	0.018	—	0.028	0.018	0.028
**8–12 Hz**	-	-	-	-	-	0.018	-	-	-	-	-	0.018
**12–30 Hz**	-	-	-	-	-	0.018	-	-	-	-	-	-
**30–48 Hz**	-	-	-	-	-	-	-	-	-	-	-	-

**Table 3:** Between-group comparisons of weighted brain network indicators.

*p*-Value	Stage 4/4*					
Frequency	PLV	aCp	aLp	aEg	aEloc	aSigma
**0.1–48 Hz**	0.048	0.035	-	-	-	-
**0.1–4 Hz**	0.018	0.011	-	-	0.017	-
**4–8 Hz**	0.048	0.006	0.013	0.015	0.048	0.048
**8–12 Hz**		-	-	-	-	0.018
**12–30 Hz**		-	-	-	-	-
**30–48 Hz**		-	-	-	-	-

## Data Availability

The datasets obtained during the current study are available from the corresponding author on reasonable request.
